# Comparison of the Efficacy of Surgical Decompression Alone and Combined With Canine Adipose Tissue-Derived Stem Cell Transplantation in Dogs With Acute Thoracolumbar Disk Disease and Spinal Cord Injury

**DOI:** 10.3389/fvets.2019.00383

**Published:** 2019-11-08

**Authors:** Fernando Swiech Bach, Carmen Lucia Kuniyoshi Rebelatto, Leticia Fracaro, Alexandra Cristina Senegaglia, Felipe Yukio Ishikawa Fragoso, Debora Regina Daga, Paulo Roberto Slud Brofman, Claudia Turra Pimpão, Jair Rodini Engracia Filho, Fabiano Montiani-Ferreira, José Ademar Villanova

**Affiliations:** ^1^Department of Neurology, Clinivet Veterinary Hospital, Curitiba, Brazil; ^2^Nucleus of Cellular Technology, Pontifical Catholic University of Paraná, Curitiba, Brazil; ^3^Postgraduate Program in Animal Science, Pontifical Catholic University of Paraná, Curitiba, Brazil; ^4^Graduate Program in Veterinary Science, Universidade Federal Do Paraná, Curitiba, Brazil

**Keywords:** dogs, paraplegia, spinal cord, neurosurgery, cell therapy, stem cells

## Abstract

Paraparesis and paraplegia are common conditions in dogs, most often caused by a disc herniation in the thoracolumbar spinal segments (T3-L3), which is a neurological emergency. Surgical decompression should be performed as soon as possible when spinal compression is revealed by myelography, computed tomography, or magnetic resonance imaging. Mesenchymal stem-cell therapy is a promising adjunct treatment for spinal cord injury. This study sought to compare the effects of surgical decompression alone and combined with an allogeneic transplantation of canine adipose tissue-derived mesenchymal stem cells (cAd-MSCs) in the treatment of dogs with acute paraplegia. Twenty-two adult dogs of different breeds with acute paraplegia resulting from a Hansen type I disc herniation in the thoracolumbar region (T3-L3) were evaluated using computed tomography. All dogs had grade IV or V lesions and underwent surgery within 7 days after symptom onset. They were randomly assigned into two groups, 11 dogs in each. The dogs in Group I underwent hemilaminectomy, and those in Group II underwent hemilaminectomy and cAd-MSC epidural transplantation. In both groups, all dogs with grade IV lesions recovered locomotion. The median locomotion recovery period was 7 days for Group II and 21 days for Group I, and this difference was statistically significant (*p* < 0.05). Moreover, the median length of hospitalization after the surgery was statistically different between the two groups (Group I, 4 days; Group II, 3 days; *p* < 0.05). There were no statistically significant between-group differences regarding the number of animals with grade IV or V lesions that recovered locomotion and nociception. In conclusion, compared with surgical decompression alone, the use of epidural cAd-MSC transplantation with surgical decompression may contribute to faster locomotor recovery in dogs with acute paraplegia and reduce the length of post-surgery hospitalization.

## Introduction

One of the most common causes of neurological disease in dogs is spinal compression secondary to disc disease, particularly in the thoracolumbar segments (T3-L3). Spinal compression results in pain, ataxia, partial or total loss of motor function, neurogenic bladder, and loss of nociception (1, 2). Spinal cord injury (SCI) may result from extrinsic factors, such as falls, fights, kicks, or ballistic projectiles, which can cause fractures and vertebral luxations, or may be secondary to intrinsic factors, such as neoplasms, fibrocartilaginous emboli, or disc herniations (1, 2).

Primary SCI occurs at the time of trauma and ranges from a small injury, caused by a minimal disc herniation into the vertebral canal, to large contusions and a severe spinal cord compression. In some animals, small amounts of herniated disc material can cause large spinal cord lesions following high-speed blunt-force trauma (2, 3). Secondary lesions may occur within hours to days after the primary lesion. These lesions are associated with a destructive cascade triggering systemic, local, and cellular events that progress to ischemia, hypoxia, edema, and various biochemical events that damage the spinal cord (4).

No effective pharmacological therapy exists for primary SCIs, and its efficacy for secondary SCIs is questionable (5). In the veterinary literature, there are several classification systems for grading SCI severity. The most commonly used classification system was proposed by Scott and McKee (6). This system identifies five grades of thoracolumbar SCI, as presented in Table 1.

**Table 1:** Grades of thoracolumbar spinal cord injury.

**Grade**	**Clinical signs**
1	Normal
2	Paraparesis, walking
3	Paraparesis, not walking
4	Paraplegia
5	Paraplegia with loss of deep pain sensation

Most authors agree that animals with grade II, III, IV, or V lesions should undergo imaging examinations, such as myelography, computed tomography (CT), or magnetic resonance imaging (MRI). If disc herniation and spinal cord compression are visualized, the animal should undergo a surgical decompression. Surgery is indicated in dogs with paraplegia because it has been determined that dogs with paraplegia that underwent hemilaminectomy had a higher proportion of locomotion recovery and a shorter time to recovery than those that underwent conservative treatment (7, 8). Surgical procedures include fenestration, hemilaminectomy, mini-hemilaminectomy, pediculectomy, and dorsal laminectomy. Hemilaminectomy has been reported to have several advantages over dorsal laminectomy, including improved access to extruded material, easy access for local fenestration and greater biomechanical stability (7, 8).

There is a direct correlation between the degree of impairment, as assessed during the neurological examination of dogs with thoracolumbar lesions, and the functional recovery. This means that dogs with grade II, III, or IV lesions exhibit a better recovery than those with grade V lesions. The timely and appropriate use of diagnostic neuroimaging and earlier surgical intervention will lower the likelihood of secondary lesion development, which will directly influence the prognosis (7).

The use of corticosteroids is not a substitute for surgical decompression; however, corticosteroids are widely used for SCI in human and veterinary medicine (7). The effect of an acute SCI due to disc herniation is reduced blood circulation in the affected area and subsequent free radical formation. Free radicals damage the spinal cord via the lipid peroxidation process, which can potentially be inhibited by corticosteroids. Compared with dexamethasone, methylprednisolone sodium succinate (MPSS) has a greater inhibitory effect on lipid peroxidation (9). Moreover, corticosteroids are effective in reducing inflammation and vasogenic edema. However, the use of corticosteroids may increase the risk of adverse effects, such as pneumonia, gastrointestinal perforation, emesis, sepsis, and death (9). Park et al. (5) reported that the use of corticosteroids to minimize the damage secondary to SCI remains controversial in veterinary medicine, particularly the use of MPSS.

Adult stem cells, such as the mesenchymal stem cells (MSCs), provide a renewable source of cells that assist in repairing the damage from various diseases or lesions (10) such as an acute or chronic SCI (11–13). Canine adipose tissue-derived MSCs (cAd-MSCs) have the potential to differentiate into other cell types, exhibit low immunogenicity, can be used for allogeneic transplantation, and do not require compatibility tests. These stem cells have tropism for damaged tissue and exert paracrine and immunomodulatory actions on the immune system cells, which can aid SCI recovery. These cells also have homing properties and move toward injured tissues. These characteristics make the cAd-MSCs a promising alternative treatment for patients with SCI (14, 15).

The utilization of stem cells in the treatment of neuropathies has been reported; angiogenesis, neurogenesis, and synaptogenesis were demonstrated after the stem-cell transplantation (16, 17).

Many studies have investigated stem-cell therapy in animals with SCI with promising results (18); however, therapeutic attempts in humans have produced little clinical improvement (19). Nonetheless, it is hard to compare these studies because they used different types and numbers of cells, as well as different routes of administration.

The present study sought to evaluate the efficacy of allogeneic cAd-MSCs as an adjuvant treatment to surgical decompression by hemilaminectomy in dogs with an acute SCI of grades IV or V secondary to disc herniation at levels T3-L3. We aimed to determine whether the transplant of epidural allogeneic cAd-MSCs could aid SCI recovery in these dogs.

## Materials and Methods

### Inclusion Criteria

**Figure 1:**
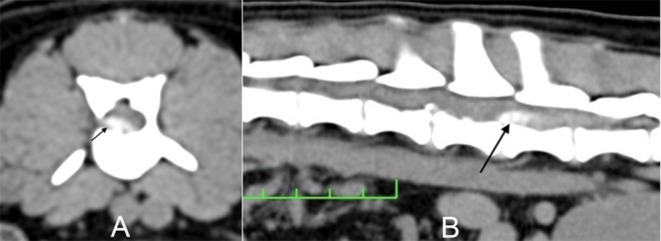
Dog 3/Group I. **(A)** Transverse computed tomography (CT) image at the level of L2-L3. **(B)** Longitudinal CT image. The arrow indicates the herniated material causing spinal cord compression.

The study protocol was approved by the Ethics Committee on the Use of Animals at the Pontifical Catholic University of Paraná (PUCPR; Curitiba, Paraná, Brazil). All of the dogs' owners signed an informed consent form for the animal's participation in the study. Adult dogs of any age or sex were recruited from the neurology department of the Clinivet Veterinary Hospital (Curitiba, Paraná, Brazil). The inclusion criteria were grade IV or V neurological lesions based on the criteria of Scott and McKee (6) (Table 1), as well as normal blood count, glucose, urea, creatinine, and alanine aminotransferase levels based on the reference values cited by Thrall (20). These dogs had an acute thoracolumbar SCI secondary to a Hansen type I disc herniation that was confirmed by CT (Figure 1). In dogs older than 7 years, the preoperative examinations included chest radiography and abdominal ultrasonography, which were used to identify potential concomitant diseases that could contribute to a poor patient recovery after spinal surgery. All included animals were deemed healthy enough to undergo surgical decompression by hemilaminectomy. Twenty-two surgeries were performed within 7 days after the onset of paraplegia.

### Groups

Twenty-two dogs with paraplegia were randomized and assigned to two groups (each group, *n* = 11). The first 11 dogs were assigned to Group I and only underwent surgical decompression by hemilaminectomy. The following 11 dogs were assigned to Group II and underwent the same procedure as Group I, as well as a transplantation of 1 ×10^7^ cAd-MSCs during surgery. The cAd-MSCs were applied in the epidural space at the level of the SCI on the surgical site. This protocol was based on the order of arrival, which allows for systematic sampling that does not cause an experimental error because the animals were independent of and had no relationship with each other. In addition, this protocol facilitated the project logistics by decreasing the expenses related to obtaining, isolating, culturing, cryopreserving, thawing, differentiating, and immunophenotypically characterizing the cAd-MSCs.

The body condition scoring (BCS) system (Table 2) was used to determine the difference in the number of overweight dogs between Group I and Group II and if this factor could influence locomotion recovery (21).

**Table 2:** Body conformation scoring (BCS) system for dogs.

**Score**	**Features**
BCS 1—Thin	Ribs, lumbar vertebrae, and pelvic bones visible at a distance and felt without pressure. No palpable fat over tail base, spine, or ribs. Diminished muscle mass. Extreme concave abdominal tuck when viewed from side. Severe hourglass shape when viewed from above.
BCS 2—Underweight	Ribs palpable with little pressure; may be visible. Minimal palpable fat over ribs, spine, or tail base. Increased concave abdominal tuck when viewed from side. Marked hourglass shape to waist when viewed from above.
BCS 3—Ideal	Ribs and spine palpable with slight pressure but not visible; no excess fat covering. Ribs can be seen with motion of the dog. Good muscle tone apparent. Concave abdominal tuck when viewed from side. Hourglass shape to waist when viewed from above.
BCS 4—Overweight	Ribs palpable with increased pressure; not visible and have an excess fat covering. Ribs not seen with motion of the dog. General hefty appearance. Abdominal concave tuck is reduced or absent when viewed from the side. Loss of hourglass shape to waist, with back slightly broadened when viewed from above.
BCS 5—Obese	Ribs and spine not palpable under a heavy fat covering. Fat deposits visible over the lumbar area, tail base, and spine. Loss of hourglass shape to waist. Complete loss of abdominal tuck, with a rounded abdomen. Back is markedly broadened.

*BCS, Body Condition Score*.

### Locomotor and Sensory Evaluation

In all of the animals, locomotion was evaluated on a rough floor by a veterinary neurologist who was unaware of the group assignment and had no contact with the owners.

Paraplegia, or grade IV, was considered to be the total absence of motor activity in the pelvic limbs, even when the dog was raised by the base of the tail. The sensory evaluation was based on the presence or absence of nociception in the proximal phalanx of the fourth toe of the pelvic limbs. A non-dentate hemostatic forceps was used to compress the phalanx to establish the presence of deep pain sensation. Limb movement (i.e., flexor reflex) was insufficient proof of deep pain. The manifestation of deep pain required body rotation and vocalization in response to the painful stimulus. The absence of deep pain sensation was a determining factor for the classification of a grade V injury.

### Isolation and Culture of the cAd-MSCs

Adipose tissue was obtained from four young and healthy female dogs that underwent elective ovariohysterectomy at the PUCPR (Curitiba, Paraná, Brazil). All samples were collected after the dogs' owners completed consent forms. The cAd-MSCs were isolated using the enzymatic digestion method described by Rebelatto et al. (22). In brief, the adipose tissue that came from the greater omentum was washed with phosphate-buffered saline solution (PBS) (Gibco; Thermo Fisher Scientific, Grand Island, NY, USA) and then digested using 1 mg/mL collagenase type I (Gibco; Thermo Fisher Scientific) for 30 min at 37°C, followed by 100 μm-filter filtration (BD Biosciences Discovery Labware, Bedford, MA, USA). The cell suspension was centrifuged at 800 g for 10 min, and the contaminating erythrocytes were removed using a lysis buffer. The cells were washed, counted, and plated at 1 ×10^5^ cells/cm^2^ in 75-cm^2^ culture flasks containing Dulbecco's Modified Eagle Medium: Nutrient Mixture F-12 (DMEM-F12) (Gibco; Thermo Fisher Scientific), which was supplemented with 10% fetal bovine serum (FBS) (Sigma-Aldrich, Saint Louis, MO, USA) and 1% antibiotics (Sigma-Aldrich). The cells were maintained at 37°C in a 5% carbon dioxide incubator, and the medium was changed twice weekly. When the cells were confluent, they were dissociated using 0.25% trypsin-ethylenediaminetetraacetic acid (EDTA) (Gibco; Thermo Fisher Scientific) and replated (Passage 1). Cells from different donors were expanded up to the third and fifth passages.

### Cryopreservation and Thawing of the cAd-MSCs

Cells were maintained until 90% confluence was reached. They were detached using trypsin-EDTA and then cryopreserved in a freezing medium (90% FBS + 10% dimethyl sulfoxide) in liquid nitrogen. On the day of transplantation, the cells were thawed, centrifuged for 10 min at 400 g, and resuspended in DMEM-F12. The cell count and viability were assessed in a Neubauer chamber. The cellular viability was evaluated by using trypan blue dye.

### Differentiation of the cAd-MSCs

The cells in the culture showed rapid cell expansion and a fibroblastoid morphology and differentiated into adipocytes, osteoblasts, and chondrocytes.

### Immunophenotypic Characterization of the cAd-MSCs

Flow cytometry was used for immunophenotypic characterization. The antibody markers used were CD45 FITC, CD44 Alexa Fluor 488, CD90 PE, CD29 PE, CD34 PE, CD9 RPE, CD14 APC, and CD8a PerCP (Table 3). The cells were washed with PBS and incubated with the antibodies in the dark for 30 min at 22–24°C. After incubation, the cells were washed with PBS and resuspended in 500 μL of 1% formaldehyde solution (Labmaster; Biotec, Paraná, Brazil). Isotypic IgG1 mouse antibodies were used as controls (BD Pharmingen, San Jose, CA, USA). Approximately 100,000 labeled cells were analyzed using the FACSCalibur flow cytometer (Becton Dickinson, Franklin Lakes, NJ, USA) and FlowJo software (FlowJo, Ashland, OR, USA).

**Table 3:** Cell surface antibodies used in the flow cytometry.

**Marker**	**Reactivity**	**Clonality**	**Brand**	**Catalog**
CD8a-PerCP	Canine	Monoclonal	eBioscience	46-5080-42
CD9-RPE	Canine	Monoclonal	AbD Serotec	MCA469PET
CD14-APC	Human. Dog: tested in development.	Monoclonal	BD Pharmingen	555399
CD29-PE	Canine	Monoclonal	Abcam	ab64629
CD34-PE	Canine	Monoclonal	eBioscience	12-0340-42
CD44-Alexa Fluor 488	Canine	Monoclonal	AbD Serotec	MCA1041A488
CD45-FITC	Canine	Monoclonal	eBioscience	11-5450-42
CD90-PE	Human. Dog: tested in development.	Monoclonal	BD Pharmingen	555596

*Manufacturer information is as follows: Abcam (Cambridge, United Kingdom), AbD Serotec (Bio-Rad, Hercules, CA, USA), BD Pharmingen (San Jose, CA, USA), and eBioscience (Thermo Fisher, San Diego, CA, USA)*.

### Anesthetic and Surgical Procedures

**Figure 2:**
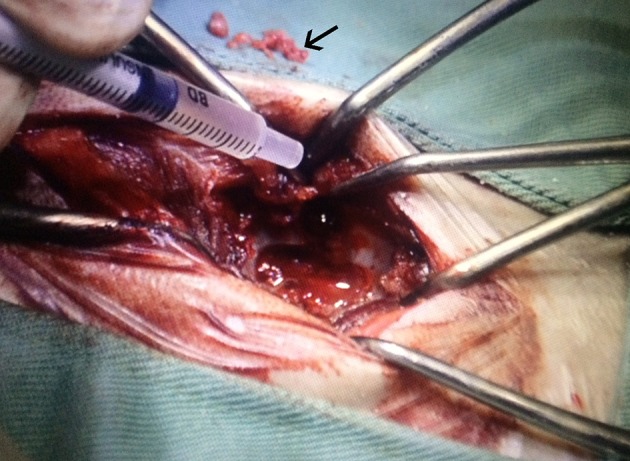
Transplantation of allogeneic cAd-MSCs after removing the disc material (arrow) causing the canine SCI. cAd-MSCs, canine adipose tissue-derived mesenchymal stem cells; SCI, spinal cord injury.

For pre-anesthesia, fentanyl citrate (5 μg/kg) and ketamine hydrochloride (1 mg/kg) were administered intravenously, followed by a bolus of propofol (5 mg/kg). Anesthesia was maintained using inhaled isoflurane and fentanyl at a dose of 10 μg/kg/h with continuous intravenous infusion. Asepsis was ensured by performing a wide trichotomy in the dorsal region, followed by antisepsis with polyvinylpyrrolidone and placement of an adhesive incisional film (Opsite Incise; Smith and Nephew, London, United Kingdom) on the surgical field. The same surgeon (who has worked in the field of small animal neurology for 13 years and has performed more than a thousand spinal surgeries) used the Sharp and Wheeler technique (7) to perform a hemilaminectomy on 22 dogs in sternal decubitus. In group II, shortly after the surgical decompression, 1 ×10^7^ cAd-MSCs were diluted in a syringe with 500 μL of vehicle DMEM-F12 between the third and fifth passages and transplanted in the epidural space around the spinal cord lesion (Figure 2). The temperature of the stem cells only at the time of transplantation was between 20° and 23°C, the same temperature of the surgery room.

### Postoperative Period

The dogs were observed for 48 h in a semi-intensive therapy unit (SITU). During this period, they received analgesia via a continuous intravenous infusion of fentanyl at a dose of 2 μg/kg/h. In addition, tramadol hydrochloride and sodium dipyrone were administered subcutaneously at 2 and 30 mg/kg, respectively, three times per day, for 5 days. Prophylactic antibiotic therapy was administered by intramuscular or intravenous injections of enrofloxacin at a dose of 5 mg/kg, once per day.

All 22 dogs were kept inside the hospital internment unit in individual comfortable cages with controlled ambient temperature, feeding, and water supply.

Each dog usually stayed 2 days in the SITU and 1 day in the normal internment unit before hospital discharge. At the time of discharge, the dogs' owners were provided guidelines on resting, urinary function monitoring, and light physiotherapy exercises. We reevaluated the dogs at 7, 14, 28, 60, and 90 days after discharge.

### Hospital Discharge Criteria

The following were the hospital discharge criteria: (1) consciousness, (2) ability to eat, (3) ability to urinate without the use of a urinary catheter, (4) established pain control, (5) a non-secreting surgical scar, and (6) scheduled to return in seven days for the first reassessment and stitch removal.

### Statistical Analysis

Since this was a clinical study on real patients, both treatment groups naturally exhibited a certain degree of heterogeneity. In order to determine the initial potential significant differences and imbalances between the two groups, different tests were used according to the type and distribution of each variable, which were analyzed using the Shapiro–Wilk test (a test of normality). The age, BCS scores, number of days from symptom onset to the surgery, days to locomotion recovery and hospitalization time were compared using Mann–Whitney *U*-tests, whereas sex and lesion grades were compared using the Chi-Square test. For data not normally distributed, the median was used as a central tendency indicator and the interquartile range (Q3-Q1) as a dispersion indicator.

Subsequently, in search of factors significantly influencing the outcome of the groups, multiple regression analysis was performed *(p* < 0.05) to compare factors such as age, locomotion recovery, and hospitalization time. Spearman's correlation was applied to analyze BCS and locomotion recovery (yes or no). All statistical analyses were performed using Statview version 5.0 (SAS Institute Inc., Cary, NC, USA).

## Results

The breed, sex, age, lesion grade and location, time to surgical decompression, locomotion recovery, length of hospitalization, and BCS score data are presented in Tables 4, 5.

**Table 4:** Group I—Characteristics of dogs that underwent surgical decompression.

**Dogs**	**Breed, sex, and age (years)**	**Lesion grade/site**	**Time to surgery/recovery (days)**	**Days of hospitalization/BCS**
1	Lhasa Apso, M, 4	IV/T13-L1	7/27	4/4
2	Cocker Spaniel, F, 8	IV/T13-L1	7/14	3/4
3	Mixed-breed, F, 7	V/L2-L3	2/28	4/3
4	Dachshund, M, 5	V/L1-L2 & L5-L6	3/No	5/5
5	Dachshund, M, 9	V/T12-T13	7/No	5/3
6	Mixed-breed, F, 8	IV/T13-L1	3/14	3/3
7	Dachshund, F, 8	V/T12-T13 & T13-L1	7/No	5/2
8	Lhasa Apso, M, 7	V/T9-T10 & T10-T11	2/No	Death/3 (myelomalacia)
9	Dachshund, M, 6	IV/T11-T12	7/28	4/3
10	Beagle, F, 7	IV/L1-L2	5/14	4/4
11	Beagle, F, 7	IV/T13-L1	5/21	5/2

*BCS, body condition score*.

**Table 5:** Group II—Characteristics of dogs that underwent surgical decompression and cAd-MSC transplantation.

**Dog**	**Breed, sex, and age (years)**	**Lesion grade/site**	**Time to surgery/recovery (days)**	**Days of hospitalization/BCS**
1	Lhasa Apso, F, 6	IV/L1-L2	2/7	3/3
2	Dachshund, M, 6	IV/T10-T11	7/3	3/3
3	Lhasa Apso, M, 4	IV/L2-L3	2/10	4/3
4	Dachshund, F, 6	IV/L2-L3	2/7	5/4
5	Lhasa Apso, F, 11	IV/T13-L1	7/28	3/3
6	Dachshund, M, 6	IV/T13-L1	1/7	3/2
7	Dachshund, M, 13	V/T13-L1	3/No	Death/5
8	French Bulldog, M, 3	V/L3-L4	3/No	3/3
9	Dachshund, F, 6	IV/T13-L1	7/7	2/4
10	Dachshund, F, 4	IV/T12-T13	7/7	3/3
11	Dachshund, M, 4	V/L2-L3	5/No	3/2

*BCS, body condition score. cAd-MSC, canine adipose tissue-derived mesenchymal stem cell*.

Table 4 presents the Group I data. This group consisted of 11 dogs, six male and five female, including four Dachshunds, two Lhasa Apsos, two Beagles, two mixed-breed dogs, and one Cocker Spaniel. Six animals had grade IV lesions, and five animals had grade V lesions. All animals with grade IV lesions recovered locomotion, whereas only one animal with a grade V injury recovered its ability to walk.

Table 5 presents the Group II data. This group consisted of 11 dogs, seven male and four female, including seven Dachshunds, three Lhasa Apsos, and one French Bulldog. Eight animals had grade IV lesions, and three animals had grade V lesions. All animals with grade IV lesions recovered locomotion, and one of the animals with grade V lesions recovered their ability to walk.

In this study, the most common site of disc herniation was the T13-L1 level (40.9%, 9/22).

Although this study was performed in real patients, the groups demonstrated homogeneity regarding the age, proportion of females and males, BCS score, proportion of lesion grades, and days to surgery, as we can prove statistically. Therefore, these factors did not influence the results.

There was no significant difference between the groups regarding age; the median age was 7.0 IQR 1.75 years in Group I, and 6.0 IQR 2.0 years in Group II (*p* = 0.11) according to the Mann–Whitney *U*-test.

In the multiple regression analysis, age was not a significant factor for either days to recovery (*p* = 0.19; *F*-value = 1.87) or hospitalization days (*p* = 0.58; *F*-value = 0.30).

The BCS was not significantly different between the groups either; the median BCS was 3.0 (interquartile range [IQR] of 1) in Group I, and 3.0 (IQR of 0.75) in Group II (*p* = 0.40) using the Mann-Whitney U Test. Of the 22 dogs, none were thin (BCS 1), four were underweight (BCS 2), 11 had an ideal weight (BCS 3), five were overweight (BCS 4), and two were obese (BCS 5). When comparing all the dogs, there was no significant correlation between BCS and locomotion recovery (Spearman's correlations = −0.015, *p* = 0.95).

Under the Chi-Square test, there was also no significant difference between the groups regarding the proportion of males and females (*p* = 0.67). No significant difference was observed regarding the proportion of Grade IV and V lesions between Groups I and II (*p* = 0.37).

There was no significant difference between groups I and II regarding the amount of time between onset of symptoms and the day of surgery; the median time was 5.0 IQR 4 days in Group I and 3.0 IQR 5 days in Group II (*p* = 0.43), according to the Mann–Whitney *U*-test.

The amount of time between the onset of clinical signs and the outcome (surgery recovery) in all dogs was 5.0 IQR 5.0 days. The median time was 3.0 IQR 3.5 days for those that never walked again and 5.0 IQR 5.0 days for those that did walk again *(p* = 0.85); therefore, they were not significantly different according to the Mann–Whitney *U*-test.

The surgical decompression alone or combined with cAd-MSCs transplantation was not a significant factor predicting locomotion recovery (*p* = 0.5) using the Chi-Square test.

**Figure 3:**
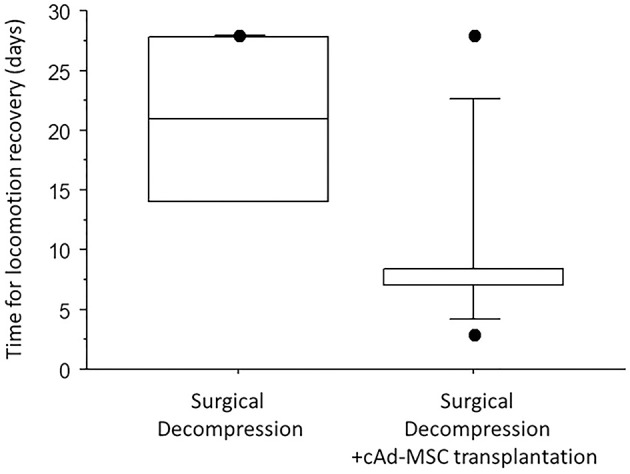
Median number of days to locomotion recovery in the two groups.

However, the time to locomotion recovery was significantly different between the two surgery groups. The dogs from Group II recovered locomotion faster than those in Group I. The median recovery times were 21.0 IQR 13.75 days in Group I and 7.0 IQR 1.5 days in Group II (*p* = 0.013) according to the Mann–Whitney *U*-test (Figure 3).

**Figure 4:**
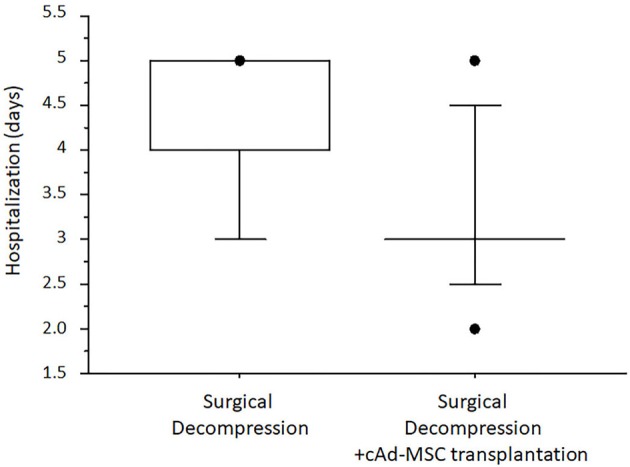
Median number of days of hospitalization after surgery.

Median hospitalization time was 4.0 IQR 1.0 days in Group I and 3.0 IQR 0.0 days in Group II. The dogs in Group II required significantly fewer days of hospitalization (*p* = 0.01) according to the Mann–Whitney *U*-test (Figure 4).

**Figure 5:**
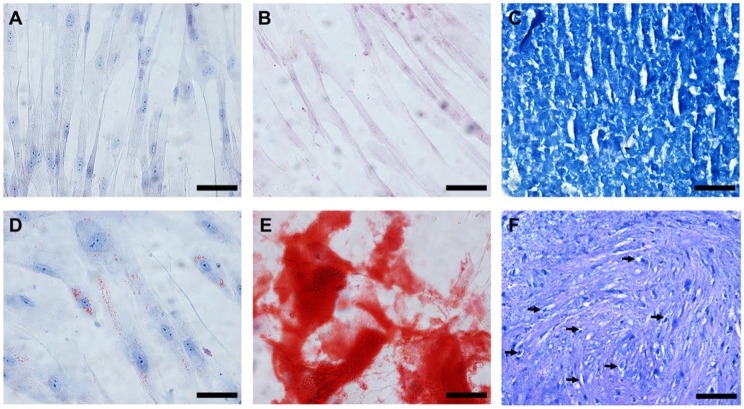
*In vitro* differentiation of the cAd-MSCs after 21 days of culture. **(A–C)** Cells cultivated with DMEM/F-12 and FBS. **(D)** Cells differentiated into adipocytes, characterized by the presence of lipidic vacuoles when stained with Oil Red O. **(E)** Cells differentiated into osteoblasts, characterized by the presence of calcium deposits when stained with Alizarin Red S. **(F)** Presence of vacuoles around young chondrocytes (arrows) and metachromatic staining with toluidine blue at the cartilaginous matrix. Scale bar: 50 μm. cAd-MSCs, canine adipose tissue-derived mesenchymal stem cells.

Regarding stem cells, the mean volume of collected adipose tissue was 65 mL, and the mean number of cells observed after isolation was 25.75 ×10^6^ cells. The cells in the culture showed rapid cell expansion and a fibroblastoid morphology and differentiated into adipocytes, osteoblasts, and chondrocytes, which confirmed that they were stem cells (Figure 5).

**Figure 6:**
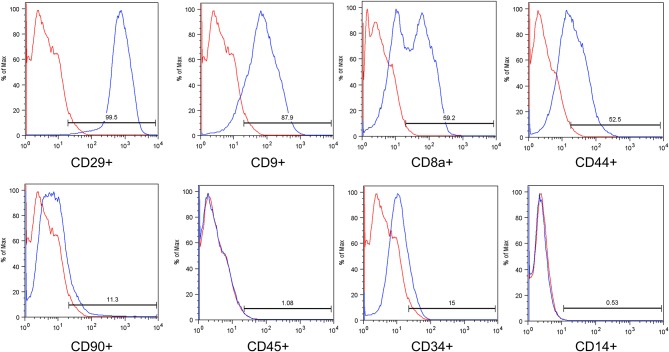
Immunophenotypic analysis of a representative sample based on flow cytometry. The blue histograms indicate the percentage of the population positive for each antibody, and the red histograms indicate the isotype controls for the antibodies.

The mean cell viability before the transplantation was 88.31%. For all samples, the microbiological tests were negative. The expression of surface antigens in the cAd-MSCs was evaluated using flow cytometry. The results were as follows: CD29 (99.5%), CD44 (52.5%), CD9 (87.9%), CD8a (59.2%), CD14 (0.53%), CD45 (1.08%), CD34 (15%), and CD90 (11.3%) (Figure 6).

## Discussion

Hansen type I disc herniation usually occurs in small dog breeds, particularly in the chondrodystrophic breeds. The Dachshund is more affected than other breeds (2). In our study, 11 of the 22 animals were Dachshunds. This condition is usually present in animals older than 2 years, with a peak incidence of the disease between 3 and 6 years of age, and there are no sex-based differences in susceptibility (2). In both groups, the mean age was slightly over 6 years, and no sex differences were observed. The main veterinary neurology reference texts do not mention any statistically significant differences in herniated disc development between animals of different weight categories. The surgical risks and the incidence of postoperative morbidity and mortality are higher in overweight dogs (2). One dog in group II that was obese died 2 days after the surgery.

Dewey and Costa (2) reported that the most common sites for thoracolumbar disc herniations are T12-T13 and T13-L1 in small dogs and L1-L2 and L2-L3 in large dogs. In this study, the most common site was T13-L1, and all of them were small and medium-sized dogs, which are predisposed to Hansen's type I disc herniation.

Compared with the Dachshund, the French Bulldog presents with herniated discs at a younger age and is more likely to develop myelomalacia. A possible explanation for this is that the French Bulldog has more material in the nucleus pulposus disk, resulting in a larger spinal compression upon herniation (23). In Group II, there was one French Bulldog with a grade V lesion that did not recover locomotion during the evaluation period of 90 days post-surgery.

Sharp and Wheeler (7) found that up to 90% of the animals with grade IV lesions that underwent surgical treatment experienced locomotor recovery, whereas Coates (1) reported that approximately 85% of such patients showed improvement. Both studies described a success rate of approximately 50% for animals with grade V lesions if they were operated on within the first 48 h after injury. If the surgery was performed after 48 h, there was a decreased success rate of 7–30%. Hu et al. (24) reported that dogs without deep pain sensation operated on before or after the first 48 h after injury have similar results in recovery. In our study, the amount of time between the onset of clinical signs and surgery was not significantly correlated to locomotion recovery. All animals with grade IV lesions in both groups recovered locomotion 100% (14/14). Of the dogs with grade V lesions, only 12.5% (1/8) recovered the ability to walk; however, the remaining patients were followed up on for only 90 postoperative days and did not recover locomotion during this time. Sharp and Wheeler (7) reported that the number of days to full locomotor recovery after hemilaminectomy ranges between 7 and 28 days in dogs with a neurological grade IV injury, and between 35 and 250 days in dogs with a grade V injury.

Animals with severe SCIs of grade V may experience locomotor recovery in 3 or more months, and the return of normal urinary function may also occur during this period. Recent studies suggested that the neuroplasticity of detrusor overactivity may continue during this period in some dogs (1, 2, 7). In our study, all dogs had voiding alterations pre-surgery and remained on a urinary catheter for the first 2 days post-surgery. After urinary catheter removal, most dogs were able to urinate normally; however, some animals required a gentle bladder massage.

Most experiments on animals with SCI involved injecting cells directly into the injured parenchyma (11). This invasive technique compromises the injured spinal cord; however, it delivers the cells into the acutely injured cord (15). Jung et al. (25) induced a SCI in dogs and observed motor improvement after an intrathecal cell transplantation. In our study, we opted to use an epidural transplantation based on the technique described by Bakshi et al. (15), because the cAd-MSCs home to the lesion site in the presence of inflammatory cytokines. As a result, it is possible to avoid injecting the cells directly into the spinal cord tissue, which can cause additional damage.

In this study, the epidural administration of cAd-MSCs at the lesion site shortly after the surgical decompression in dogs with acute SCI, that is, <7 days after injury, may have allowed the cAd-MSCs to attenuate the inflammatory response, decrease the astrocytic immunoreactivity, and activate the endogenous stem cells. We used this route because it has been confirmed using immunofluorescence analysis that the blood-brain barrier is disrupted when the spinal cord is injured, which allows the cells to migrate into the medullary parenchyma (14, 26). The author has seen that, in some cases of thoracolumbar disk herniation damage in the dura mater, this phenomenon may help the cAd-MSCs cross the dura mater into the spinal parenchyma. In extreme cases, the herniated material can penetrate the spinal cord.

Kurozumi et al. (27) and Quertainmont et al. (28) demonstrated that cAd-MSCs are capable of secreting growth factors and cytokines, such as neural growth factor and vascular endothelial growth factor, which increase the expression of anti-inflammatory cytokines such as interferon-gamma and interleukin-10. After an SCI, the trophic characteristics of cAd-MSCs may have the beneficial effect of repairing and reorganizing the neuronal connections, inducing regeneration, stimulating neurogenesis and axonal growth, reducing the inflammatory response, and protecting the tissue. However, these mechanisms are not long-lasting, potentially lasting only a few weeks, and may not affect chronic lesions with glial scar formation (29). In our study, the short period between the diagnosis and the treatment, as well as the presence of inflammatory cytokines at the lesion site, allowed the cAd-MSCs to home to the neural tissue, which allowed them to exert paracrine anti-inflammatory actions and modulate the injured environment.

The stem cells were derived from the adipose tissue of the greater omentum because of their favorable results in dogs with experimentally induced SCIs, and adipose tissue has a large number of stem cells that can be easily obtained (30, 31).

For the flow-cytometry analysis, it was not possible to conduct a panel for all MSC characteristics, as is suggested by the International Society for Cellular Therapy (Vancouver, BC, Canada), because not many species-specific antibodies exist for dogs. However, several authors have demonstrated that cAd-MSCs are positive for CD9 and CD29 markers and negative for CD45 markers, which corroborates the results of the current study (32–34).

Other markers were evaluated in the cAd-MSCs, such as the positively expressed CD29, which is an important marker for human MSC definition, and CD14 and CD34, which have reduced expression in cAd-MSCs (33, 35, 36). In this study, CD90 showed reduced expression; however, it is positively expressed in human MSCs. There is no consensus on the CD90 expression in canine MSCs. Several authors have demonstrated CD90-positive and CD90-negative expression (32–34, 36, 37). The results obtained with the set of markers used in this study demonstrate that the cells used were MSCs.

In our study, cryopreserved cells that were thawed before transplantation were used. Some studies have immunophenotyped and evaluated the proliferative and differentiative capacity of the MSCs before and after the cryopreservation. Yong et al. (38) and Gonzales-Fernandez et al. (39) demonstrated that after cryopreservation, human and mouse MSCs retain their characteristics and maintain stemness, a typical characteristic of MSCs. Martinello et al. (40) used the same source and cryopreservation protocol used in this study and demonstrated that the potential for differentiation and surface marker expression is maintained after cryopreservation.

The results of this study suggested that MSCs used after cryopreservation or thawing continue to have a greater potential for cell differentiation than control cells and maintain the same cell surface antigen and stemness profile. These characteristics demonstrate the utility of MSCs in clinical practice.

Our study has some limitations. Langerhuus and Miles (8) have seen some dogs with a grade V injury recover faster than dogs with a grade IV injury; this is not common, but it can happen in some dogs. Therefore, it cannot be stated with certainty that the cAd-MSCs will aid SCI recovery based solely on the time to recovery, as individual factors can influence the time to recovery. In contrast, it can be stated that surgical decompression is better than conservative treatment because a greater proportion of dogs recovered locomotion and the recovery to ambulation was faster in the dogs treated with hemilaminectomy than in those treated conservatively.

We evaluated the efficacy of stem cells in this study by comparing the effect of surgical and combined treatment. There was a significant difference between group I and group II in the time to locomotion recovery and time of hospitalization; however, no between-group difference was found in the recovery itself.

Another limitation of this study is that no histopathological examination was performed; Jung et al. (25) conducted histopathological examinations of the spinal cords of dogs with induced thoracolumbar lesions after euthanasia and showed that significant differences existed between the control and stem cell groups after 5 weeks. The stem cell group had a lower volume of myelomalacia and minimal intramedullary cavitations. In our study, it was not possible to perform histopathology because the animals were not laboratory subjects, the lesions were not induced, and the owners were expecting recovery. The animals were treated at a private veterinary hospital.

MRI is an imaging technique that is superior to CT for the evaluation of the medullary parenchyma (1). In future investigations, MRI should be conducted to monitor medullary parenchyma changes before surgical decompression, immediately afterwards, and during the late postoperative period (i.e., 5 weeks). This schedule was suggested by Jung et al. (25), who studied Beagles with induced spinal cord injuries. These authors found no MRI differences between the control and stem cell groups during the first week; however, after 5 weeks, the lesion size was significantly reduced in the stem cell-treated dogs compared with the control dogs.

## Conclusions

The epidural transplantation of cAd-MSCs at the time of decompression surgery can positively contribute to motor improvement in dogs with acute disk herniation and paraplegia. Compared with dogs that underwent surgical decompression alone, those that underwent combined treatment with cAd-MSCs had shorter locomotion recovery and hospitalization periods.

## Data Availability Statement

All datasets generated for this study are included in the article/supplementary material.

## Ethics Statement

This study protocol was approved by the Ethics Committee on the Use of Animals at the Pontifical 107 Catholic University of Paraná (PUCPR; Curitiba, Paraná, Brazil). All dogs' owners signed an informed 108 consent form for the animals' participation in the study.

## Author Contributions

FB performed all computed tomography and surgeries. CR, LF, AS, FF, DD, and PB were responsible for the stem-cells. JE and CP helped to elaborate the manuscript. FM-F was responsible for the statistical analysis. JV helped in the orientation and wrote the manuscript.

### Conflict of Interest

The authors declare that the research was conducted in the absence of any commercial or financial relationships that could be construed as a potential conflict of interest.
